# Dynamic Discordance Between Creatinine- and Cystatin C-Based Estimated Glomerular Filtration Rates Across Forniceal Rupture and Its Surgical Decompression: A Case Report

**DOI:** 10.7759/cureus.113573

**Published:** 2026-07-29

**Authors:** Donghui Kim, Hyun Cheol Jeong

**Affiliations:** 1 Urology, Kangdong Sacred Heart Hospital, Hallym University College of Medicine, Seoul, KOR

**Keywords:** creatinine, cystatin c, forniceal rupture, glomerular filtration rate, pseudoazotemia, ureteral calculi, urinary extravasation

## Abstract

Forniceal rupture from an obstructing ureteral stone can raise serum creatinine, but whether that rise reflects a true fall in filtration or pseudoazotemia from reabsorbed extravasated creatinine is easily missed. We describe a woman in her 70s with calculous forniceal rupture and retroperitoneal urine leakage in whom serial creatinine and cystatin C, together with the difference between their estimated glomerular filtration rates (ΔeGFR, cystatin C-based minus creatinine-based), were tracked over the course of the illness. The gap was large and positive at presentation (+41.7 mL/min/1.73 m²), reversed to a negative nadir on the operative morning (−7.4), when a creatinine that had fallen only to the low-normal range might, on its own, have been mistaken for a recovery that the lower cystatin C-based estimate did not support. It then returned through postoperative day 1 (−2.7) to a modest positive value by day 8 (+7.6), well below the presenting gap though not resolved to zero. Over time, the direction of this discordance helped separate reabsorptive pseudoazotemia from a within-range creatinine that alone would have overstated recovery.

## Introduction

Forniceal rupture, the spontaneous disruption of the renal fornix, is usually precipitated by acute calculous ureteral obstruction, with urine escaping into the retroperitoneal space. It often settles once the obstruction is relieved, yet it may be accompanied by an apparent rise in serum creatinine: in a prospective observational series of 40 such patients, 12 (30%) had elevated creatinine, but a genuine fall in glomerular filtration rate (GFR) was not distinguished from reabsorption of the extravasated urinary creatinine [1].

The apparent azotemia turns on molecular size. Creatinine is a small molecule (113 Da) that is reabsorbed from the extravasated urine pool into the bloodstream, while the much larger cystatin C (about 13 kDa) stays behind; it is this reabsorbed creatinine, not a true loss of filtration, that raises the serum creatinine, at times only within its reference range, as in the present case. This size-selective transfer is well characterized for the peritoneum, and we extend it here by analogy to the retroperitoneal surface - an inference rather than a process directly demonstrated in this patient [2]. Creatinine-based estimated GFR (eGFR) is therefore artifactually depressed, even when the serum creatinine remains within its reference range, while the cystatin C-based estimate still tracks true renal function, and the difference between them (ΔeGFR) becomes measurable [3].

Cystatin C has been used to expose this pseudoazotemia before, but in other anatomical settings and at a single timepoint [4-6]. Our targeted PubMed search (Appendix A) returned no account of the discordance in forniceal rupture caused by an obstructing ureteral stone with retroperitoneal extravasation, nor of how it evolves across obstruction and surgical relief. We present a woman in her 70s in whom serial ΔeGFR traced a dynamic phase shift, from positive (pseudoazotemia) through a negative nadir and back toward a small positive value after ureteroscopic stone removal and stent decompression, showing how two routine markers, read over time, can help disentangle concurrent processes and follow their resolution.

## Case presentation

**Figure 1:**
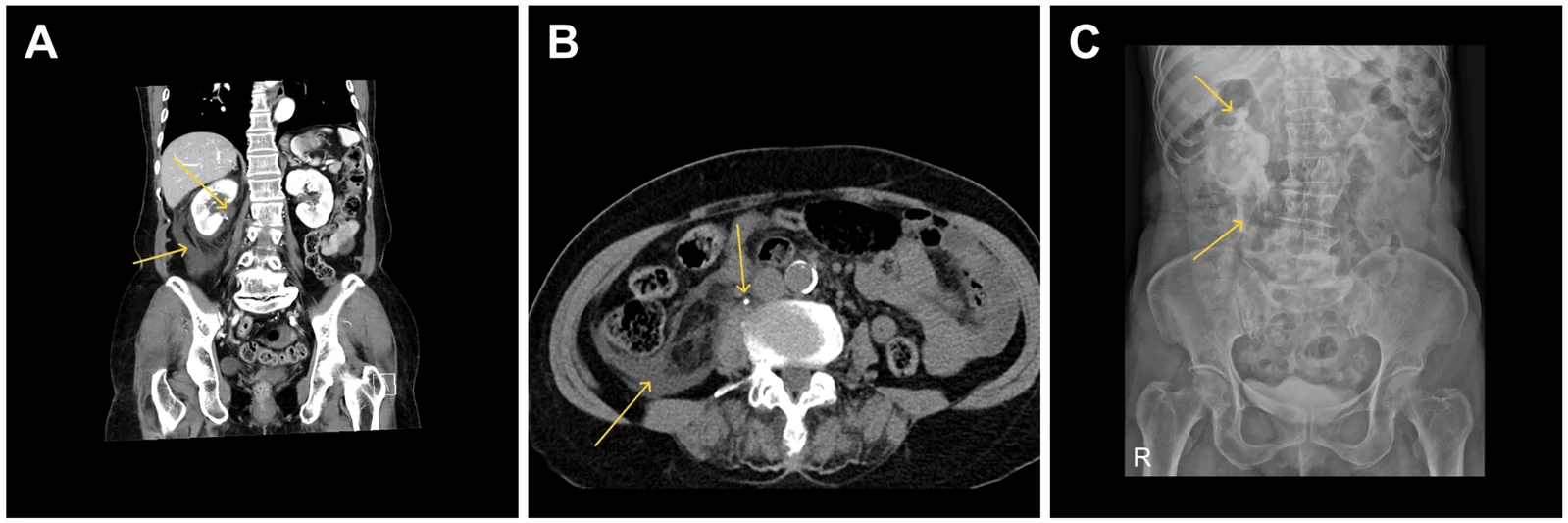
Imaging at presentation confirming forniceal rupture with retroperitoneal urine leakage. (A) Coronal contrast-enhanced computed tomography (CT) showing right hydronephrosis (upper arrow) and perirenal fluid (lower arrow). (B) Axial contrast-enhanced CT showing the obstructing right ureteral stone (upper arrow) and adjacent retroperitoneal fluid (lower arrow). (C) Post-CT plain radiograph (kidney-ureter-bladder) showing contrast-medium spillage in the right upper abdomen (arrows), the direct radiographic sign of forniceal rupture with retroperitoneal urine leakage.

A previously healthy woman in her 70s, with no history of chronic kidney disease, diabetes, or hypertension, presented to the emergency department with acute right flank pain. Contrast-enhanced computed tomography (CT) demonstrated a 5 mm stone in the right upper ureter with hydroureteronephrosis, moderate fluid collections in the right perirenal and paracolic gutter spaces, and segmental wall enhancement suggesting inflammatory change. A post-CT plain radiograph (kidney-ureter-bladder) showed the right upper ureteral stone with hydroureteronephrosis and contrast-medium spillage, confirming forniceal rupture with retroperitoneal urine leakage (Figure 1). Intravenous ceftriaxone was started immediately on recognition of the urine leak, and she was admitted.

At presentation (day 0), both serum creatinine and cystatin C were within their reference ranges, yet the creatinine-based eGFR fell well below the cystatin C-based estimate, giving a large positive discordance (ΔeGFR; Table 1). We interpreted this depressed creatinine-based estimate as reabsorptive pseudoazotemia - an artifactual lowering of the creatinine-based eGFR by reabsorbed extravasated creatinine - rather than a true loss of filtration.

**Table 1:** Serial laboratory values across forniceal rupture and its surgical decompression. ΔeGFR is defined as the cystatin C-based estimate minus the creatinine-based estimate (positive = pseudoazotemia). Samples were drawn at 18:20 on the day of presentation (day 0), at 05:27 on the operative morning (approximately 4.5 hours before ureteroscopic stone removal with stent placement, performed at 10:00), at 05:35 on postoperative day 1, and at 09:21 on postoperative day 8. Reference intervals are those of the performing clinical laboratory (creatinine measured by kinetic Jaffe). ^§^The cystatin C reference interval is an upper limit of 1.0 mg/L. *The postoperative day 8 value (1.030) is marginally above the limit. ^†^The laboratory reports the cystatin C-based (Larsson) estimate in absolute mL/min, with an age-adjusted reference lower limit of 77.2 mL/min; for commensurability with the body-surface-area-indexed creatinine-based estimate, the cystatin C-based values and this reference limit are shown here indexed to 1.73 m² (patient body surface area approximately 1.58 m², DuBois formula; indexed lower limit 84.5 mL/min/1.73 m²), a scaling that preserves each value's position relative to the limit and hence the flags. No reference lower limit is provided for the creatinine-based Chronic Kidney Disease Epidemiology Collaboration (CKD-EPI) 2009 estimate, which is therefore shown without a flag; both estimates are interpreted alongside Kidney Disease: Improving Global Outcomes (KDIGO) chronic kidney disease GFR categories. ^‡^ΔeGFR is a derived research metric without a laboratory reference range; a between-marker gap beyond ±15 mL/min/1.73 m² is regarded as clinically meaningful in population data [3]. ΔeGFR was computed from unrounded estimates and may differ by up to 0.1 mL/min/1.73 m² from subtraction of the rounded values shown. H: above the reference range; N: within the reference range; L: below the reference range; GFR: glomerular filtration rate; eGFR: estimated glomerular filtration rate

Parameter	Reference range	Presentation (day 0)	Operative morning	Postoperative day 1	Postoperative day 8
Creatinine (mg/dL)	0.6-1.2	1.06 (N)	0.78 (N)	0.81 (N)	0.78 (N)
Cystatin C (mg/L)	≤1.0^§^	0.930 (N)	1.210 (H)	1.190 (H)	1.030 (H*)
Creatinine-based eGFR (mL/min/1.73 m²)	--^†^	51.0	73.8	70.6	73.8
Cystatin C-based eGFR (mL/min/1.73 m²)	≥84.5^†^	92.6 (N)	66.5 (L)	67.9 (L)	81.5 (L)
ΔeGFR (cystatin C − creatinine)	--^‡^	+41.7	−7.4	−2.7	+7.6
Blood urea nitrogen (mg/dL)	6-26	25.2 (N)	15.5 (N)	15.2 (N)	14.5 (N)

By the morning of the operative day (approximately 4.5 hours before surgery), creatinine had fallen to the low end of its reference range, whereas cystatin C had risen above its reference range to its peak (Table 1). Correspondingly, the creatinine-based eGFR now exceeded the cystatin C-based estimate, reversing the discordance to a negative value. The falling creatinine suggested that reabsorption of extravasated urine had diminished, in keeping with resolution of the leak, while the concurrently elevated cystatin C, a marker not subject to this reabsorption, indicated that the low-normal creatinine could not yet be taken as evidence of fully recovered filtration. This reading remains inferential: resolution of the urine leak was not directly demonstrated at this timepoint, and non-steady-state marker kinetics, hydration status, or other perioperative factors may also have contributed to the change in serum creatinine.

**Figure 2:**
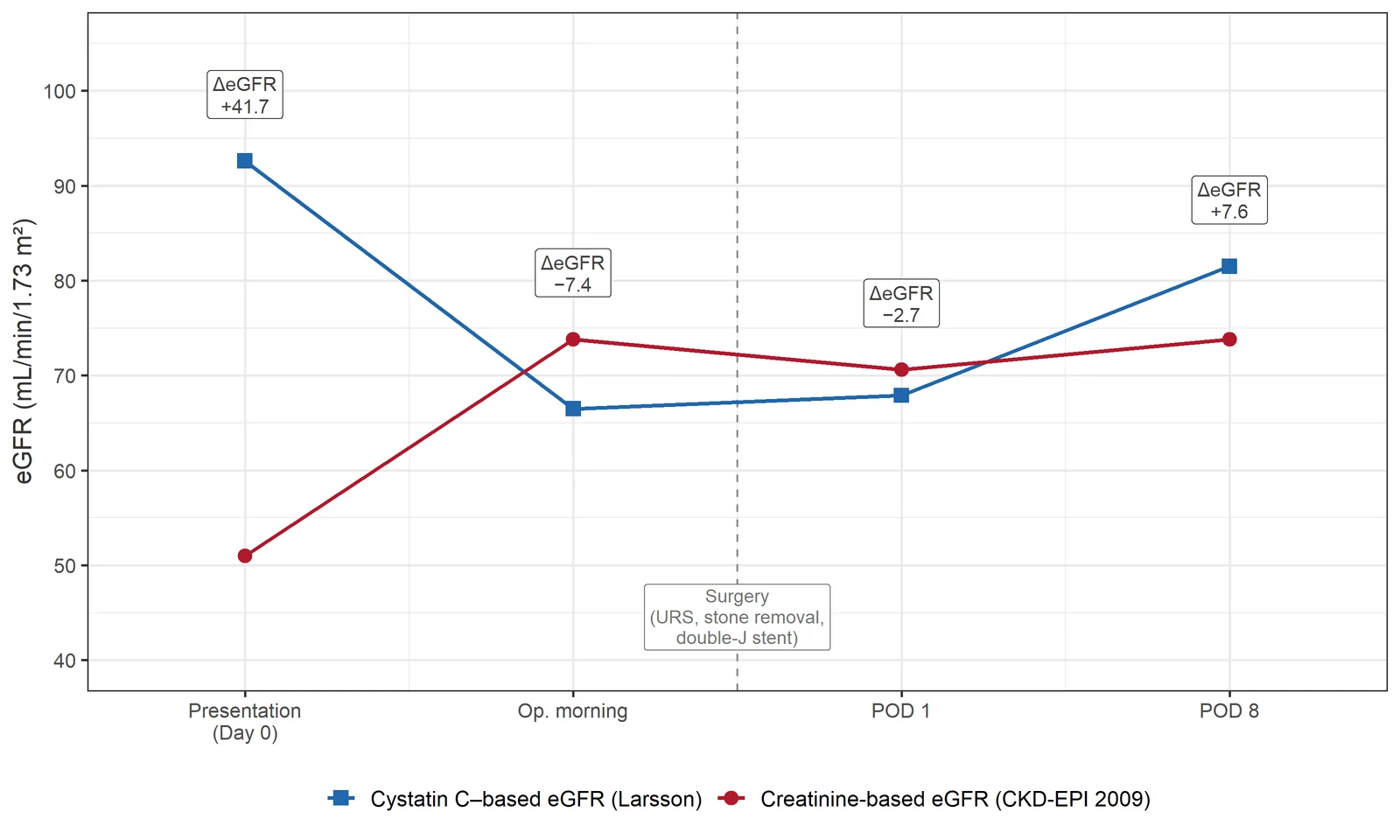
Serial creatinine- and cystatin C-based eGFR across forniceal rupture and its surgical decompression. Estimated glomerular filtration rate (eGFR, mL/min/1.73 m²) is plotted at four timepoints: presentation (day 0), the operative morning (approximately 4.5 hours before surgery), the first postoperative day (POD), and outpatient review with stent removal (POD 8). Red circles denote creatinine-based eGFR (Chronic Kidney Disease Epidemiology Collaboration (CKD-EPI) 2009 equation) and blue squares cystatin C-based eGFR (Larsson equation, indexed to 1.73 m² for commensurability). The vertical line marks ureteroscopy (URS) with complete stone removal and double-J ureteral stent placement. ΔeGFR (cystatin C-based minus creatinine-based) is annotated at each point (+41.7, −7.4, −2.7, +7.6); a positive value indicates pseudoazotemia from reabsorbed extravasated creatinine. The two estimates diverge at presentation and cross before surgery - when a within-range creatinine alone might be read as recovery. The discordance reached its negative nadir at the preoperative sample and thereafter returned to a modest positive value rather than closing; the exact turning point relative to surgery is not resolved by the available sampling.

Ureteroscopy with complete stone removal and placement of a double-J ureteral stent was performed on the operative day. On the first postoperative day, the negative discordance had begun to narrow. At an outpatient review on postoperative day 8, when the stent was removed, the patient was asymptomatic; creatinine remained within its reference range, cystatin C had improved, and the discordance had returned to a small positive value, well below its presenting level (Table 1; Figure 2).

Intravenous ceftriaxone (2 g daily) was given until discharge on postoperative day 1, then switched to oral cefpodoxime (200 mg twice daily) for seven days. Throughout the admission, she remained hemodynamically stable, with no hypotension and no signs of volume depletion or overload, and was clinically euvolemic. She received maintenance intravenous fluid alongside oral intake, with an approximately neutral daily fluid balance and non-oliguric urine output (about 3.3 L on the day before surgery). Blood pressure was transiently elevated at presentation in the setting of acute pain and had normalized by the preoperative assessment. Markers of infection stayed within normal limits throughout: the white blood cell count remained normal (with a transient neutrophil predominance), C-reactive protein remained within its reference range, and procalcitonin was below the detection limit. Urinalysis showed gross hematuria without pyuria and a negative nitrite test, and urine Gram stain and culture were negative, consistent with a sterile urinoma. Antibiotics were therefore given as prophylaxis against secondary infection of the extravasated urine rather than to treat an established infection. Blood urea nitrogen, high-normal at presentation, fell steadily thereafter (Table 1), in keeping with progressive recovery.

The serial laboratory values across the clinical course are summarized in Table 1. Written informed consent for publication of the clinical data was obtained from the patient.

## Discussion

Throughout, ΔeGFR was taken as the cystatin C-based estimate minus the creatinine-based estimate, the convention of earlier discordance work [3,7], so that a positive value means creatinine underestimates the GFR (pseudoazotemia). What stood out was not the discordance itself but its evolution: from a large positive value at presentation (+41.7), through a negative nadir on the operative morning (−7.4), and back toward a modest positive value across recovery (−2.7 on the first postoperative day, +7.6 by day 8, still well below the presenting gap). Only the presenting value exceeded the ±15 mL/min/1.73 m² population threshold; the later values all fell within that band and traced a reversal of direction rather than a monotonic convergence. Because these shifts occurred within one patient, time-invariant influences such as muscle mass or a fixed equation offset cannot explain them: a constant offset cannot change sign, yet the discordance inverted from positive to negative across the operative course; this initial reversal held whether or not the cystatin C estimate was indexed to body surface area, and under alternative equations as well (Appendix B), whereas the later return toward positive proved equation-dependent. Each phase maps onto a distinct state - creatinine reabsorption during active extravasation, a phase in which a low-normal creatinine could have falsely reassured clinicians while cystatin C had not returned to baseline, and recovery once the stone was removed and the system decompressed. Earlier reports captured the discordance at a single timepoint [4-6]; this case adds the temporal dimension.

Because a documented premorbid baseline was not available, we take the patient's stabilized postoperative values as the reference point: relative to them, the presenting creatinine was mildly higher and the creatinine-based eGFR correspondingly lower. We therefore use pseudoazotemia to denote this reabsorption-driven, artifactual depression of the creatinine-based estimate that the discordance reveals, rather than a frank elevation above the range. By day 8, the discordance had fallen well below its large presenting value but had not resolved to zero, returning to a modest positive ΔeGFR (+7.6 using commensurate, body-surface-area-indexed estimates), whose sign at this endpoint is itself equation-dependent (Appendix C). Without a premorbid baseline, this small positive value cannot be separated into a residual pseudoazotemic offset versus this patient's own inherent between-marker difference; the cystatin C-based estimate remaining just below its reference limit separately indicates that filtration had improved but not fully recovered. Together, these place the patient short of a full return to her own baseline.

On the operative morning, serum creatinine had fallen to the low-normal range - a value that, read alone, might have been taken for recovered filtration. Cystatin C moved the opposite way, rising to its peak above the reference limit, reversing the discordance and warning against that reading; at this point, cystatin C stood frankly above its reference limit and the cystatin C-based eGFR had fallen below the laboratory reference (Table 1), an objective signal independent of the magnitude of ΔeGFR. Because the contralateral kidney was unobstructed, serum creatinine stayed within the reference range, as expected in unilateral obstruction [8]; this is why cystatin C, sensitive within that range, was informative. Whether the rise reflected a genuine transient reduction in filtration or the differing non-steady-state kinetics of the two markers cannot be settled from a single case; at each step, it was the direction of the discordance, not any one value, that was clinically useful. This is the interpretive gap left by earlier work, which reported an elevated creatinine without separating a true fall in filtration from reabsorption or measuring cystatin C [1].

Our observation complements earlier work rather than displacing it. Cystatin C has unmasked pseudoazotemia in urinary extravasation before - largely intraperitoneal and at single timepoints [4-6], or, in bladder-rupture ascites, as a creatinine/cystatin C ratio that rose and resolved with drainage [9]. The present case differs in setting, metric, and trajectory: the extravasation was retroperitoneal and stone-obstructive; we followed the difference between the two eGFR estimates rather than their ratio; and the discordance did not merely rise and resolve but reversed, its negative phase warning against the false reassurance of a within-range creatinine, with surgery as the landmark after which the cystatin C-based estimate recovered. Forniceal rupture from an obstructing stone has been reported as a surgical entity, but without these markers [10].

Several features strengthen this reading. The patient had no chronic kidney disease, diabetes (a normal HbA1c), or hypertension; no corticosteroids were given; and thyroid function was normal (thyroid-stimulating hormone and free thyroxine both within range), arguing against the main non-renal influences on cystatin C. The sterile course (a normal white blood cell count, a C-reactive protein within its reference range, and a negative urine culture) also argues against an infection-driven rise in cystatin C. A prerenal contribution to the presenting creatinine is unlikely, since the cystatin C-based estimate was normal at presentation, indicating that true filtration was preserved when the creatinine-based estimate was most depressed. The near-neutral daily fluid balance likewise argues against volume depletion or a dilutional contribution to the later fall in creatinine. Because the obstruction was unilateral, the preserved, non-oliguric urine output, presumably drawn largely from the unobstructed contralateral kidney, does not exclude the modest reduction in filtration that the rising cystatin C may signal, since urine volume and glomerular filtration are not interchangeable here.

Several limitations temper these observations. This is a single case and needs prospective confirmation. For eGFR, we used the Larsson cystatin C formula [11] together with the Chronic Kidney Disease Epidemiology Collaboration (CKD-EPI) 2009 creatinine formula [12]; the Larsson estimate is reported by the laboratory in absolute mL/min, and for the primary comparison, we indexed it to 1.73 m² using the patient's body surface area so that the two estimates were dimensionally commensurate. The direction of the discordance and its reversal persisted whether the cystatin C estimate was indexed or not and across a range of alternative equation combinations (Appendix C); the day 8 endpoint, by contrast, is more sensitive to the choice of equation, so we rest our reading on the reversal and its timing rather than on the size of the residual gap. The reabsorptive capacity of the retroperitoneum relative to the peritoneum, from which the mechanism is inferred by analogy, is not directly established here. Creatinine was quantified with a kinetic Jaffe method, a technique in which certain cephalosporins can spuriously raise readings; here, though, the principal discordance was already present at presentation, before any sustained cephalosporin exposure, and creatinine fell rather than rose during treatment, the opposite of what such interference would create. The ±15 mL/min/1.73 m² figure [3] comes from steady-state cohort data and is invoked here only as a rough frame of reference, not a validated cut-off for an acute course like this one. Because the markers were not at steady state, differences in their kinetics may also contribute to the discordance; serial cystatin C-creatinine data in urinary extravasation remain scarce, so the mechanism of the negative phase awaits prospective study. Finally, because the stone was removed and the stent placed together, the separate contributions of relieving obstruction and diverting urine to recovery cannot be teased apart.

## Conclusions

Read serially across forniceal rupture and its surgical decompression, the difference between two routine markers (creatinine and cystatin C) helped separate reabsorptive pseudoazotemia from a within-range creatinine that on its own would have overstated recovery, and it tracked the reversal and recovery of this discordance over time, from a large positive value at presentation to a small one well below it. Because cystatin C can be measured on the same blood sample as creatinine, the difference between the two estimates is a simple subtraction that adds no procedure to the workup, and its direction can be followed over time.

The lesson lies not in the size of the gap but in its direction and its change: a creatinine that looks reassuring in isolation can still mislead when cystatin C has not yet returned to baseline. A single case cannot establish how generally this behaves, and a prospective study is needed; even so, tracking the two markers against each other provides an inexpensive check against over-reading recovery when a stone drives the urinary leak.

## Appendices

Appendix A

Literature Search Strategy

The scoped novelty claim in the main text - that we found no prior description of a creatinine-cystatin C eGFR discordance in forniceal rupture from an obstructing ureteral stone, nor of its evolution across obstruction and surgical decompression - rests on the search summarized here, and is stated as bounded ("within the literature identified by our PubMed search") rather than absolute.

We searched PubMed on May 30, 2026, with no language, date, or article-type filters, using 13 queries grouped by concept: extravasation and reabsorptive pseudoazotemia (for example, urinoma pseudoazotemia, urinoma "creatinine reabsorption"); pelvic, calyceal, and forniceal rupture paired with a biomarker or filtration term (for example, "forniceal rupture" cystatin, "forniceal rupture" creatinine); and cystatin C in urinary extravasation or obstruction (for example, cystatin C urinary extravasation, cystatin C urinoma).

Queries pairing forniceal or calyceal rupture directly with cystatin C returned nothing, whereas the broader extravasation queries retrieved relevant work. Cystatin C had been used to separate pseudoazotemia from true filtration loss in urinary extravasation before, but in other anatomical settings - intraperitoneal leakage of bladder or gynecologic origin [4-6] - and as single-timepoint observations. The largest forniceal rupture series identified reported elevated creatinine in 30% of patients without distinguishing genuine obstruction from reabsorptive pseudoazotemia and without cystatin C [1]. One report described pseudo-renal failure from urinary extravasation that also followed a forniceal rupture, but there the rupture was caused by invasive bladder carcinoma rather than an obstructing stone, the leak was intraperitoneal rather than retroperitoneal, it was captured at a single timepoint, and cystatin C was not measured [13]. Across these results, we found no report applying a creatinine-cystatin C eGFR discordance to obstructive forniceal rupture, or following such a discordance through obstruction and surgical decompression.

This was a single-database search; Embase, Scopus, Web of Science, Google Scholar, and the Korean databases KMbase and KoreaMed were not queried, so reports indexed only there would have been missed. The claim should therefore be read as describing the literature retrieved by this defined search.

Appendix B

Equations and Creatinine Assay

All laboratory testing was performed at the institutional central laboratory. Cystatin C-based eGFR was derived from serum cystatin C with the Larsson equation [11] (cystatin C in mg/L), namely, eGFR = 77.24 × (cystatin C)^−1.2623^. The Larsson equation returns an absolute estimate (mL/min); reverse calculation from the measured cystatin C reproduced the laboratory's values before normalization, confirming this equation as their basis. For the primary comparison in Table 1 and Figure 2, and consistently with our companion report on the same discordance, the Larsson estimate was indexed to 1.73 m² using the patient's body surface area (approximately 1.58 m² by the DuBois formula, giving an indexing factor of 1.0951) so that it was dimensionally commensurate with the body-surface-area-indexed creatinine estimate; the laboratory's age-adjusted reference lower limit for this estimate (77.2 mL/min) was indexed by the same factor (84.5 mL/min/1.73 m²), which leaves each value's position relative to the limit, and therefore the reference flags, unchanged. Creatinine-based eGFR used the Chronic Kidney Disease Epidemiology Collaboration (CKD-EPI) 2009 equation [12]; reverse calculation identified this equation, rather than alternatives, as the basis of the reported values. Serum creatinine was measured by a kinetic Jaffe (alkaline picrate) method.

Appendix C

Sensitivity of the ΔeGFR Trajectory to Equation Choice

To test whether the trajectory depended on the particular equations used, ΔeGFR was examined at the four timepoints under three creatinine-cystatin C equation combinations - the primary pairing and two alternative equation sets - taking every constant from the original publication (Table 2).

**Table 2:** Sensitivity of the ΔeGFR trajectory to the choice of creatinine-cystatin C equation. ΔeGFR in mL/min/1.73 m². The first row reproduces the primary comparison of Table 1 - the Larsson cystatin C estimate indexed to 1.73 m² using the patient's body surface area, minus the laboratory-reported CKD-EPI 2009 creatinine estimate. The remaining rows were recomputed from the measured creatinine and cystatin C with each equation's own published constants; the CKD-EPI 2012-2021 and FAS pairings return values already normalized to 1.73 m² and so require no further indexing.

Cystatin C × creatinine combination	Presentation	Operative morning	Postoperative day 1	Postoperative day 8	Direction
Larsson (indexed) − CKD-EPI 2009 (primary)	+41.7	−7.4	−2.7	+7.6	+ → − → − → +
CKD-EPI 2012 cystatin C − CKD-EPI 2021 creatinine [14,15]	+21.5	−25.1	−20.4	−12.3	+ → − → − → −
Full age spectrum (FAS) cystatin C − FAS creatinine [16]	+25.1	−7.8	−4.6	+1.7	+ → − → − → +

Across every combination, the discordance was large and positive at presentation and reversed to negative on the operative morning, the positive-to-negative shift that defines the trajectory. This reversal, the trajectory's defining feature, was therefore robust to equation choice, whether the cystatin C estimate was indexed to body surface area (Larsson) or expressed on a 1.73 m² basis by construction (CKD-EPI 2012, FAS). The postoperative day 8 endpoint, by contrast, was equation-dependent: the Larsson and FAS pairings returned a positive value (+7.6 and +1.7), whereas the CKD-EPI 2012-2021 pairing retained a moderate negative value (−12.3), reflecting that equation set's systematically lower cystatin C-based estimates. Robustness thus applies to the reversal across decompression, not to the precise endpoint.

## References

[1] Al-Mujalhem AG, Aziz MS, Sultan MF, Al-Maghraby AM, Al-Shazly MA (2017). Spontaneous forniceal rupture: can it be treated conservatively?. Urol Ann.

[2] Kabanda A, Goffin E, Bernard A, Lauwerys R, van Ypersele de Strihou C (1995). Factors influencing serum levels and peritoneal clearances of low molecular weight proteins in continuous ambulatory peritoneal dialysis. Kidney Int.

[3] Wang Y, Adingwupu OM, Shlipak MG (2023). Discordance between creatinine-based and cystatin C-based estimated GFR: interpretation according to performance compared to measured GFR. Kidney Med.

[4] Woldu SL, Matulay JT, Silva MV (2015). Serum cystatin C as a novel marker to differentiate pseudoazotemia in the setting of intraperitoneal urine extravasation. Urology.

[5] Saro-Nunez L, Aufderheide A, Baskharoun S, Perlman A (2018). Utility of cystatin C in the setting of urinoma. Ann Clin Lab Sci.

[6] Yun S, Kang M, Song SM (2026). Pseudo-renal failure with hyponatremia and proteinuria after gynecologic surgery: a case report with literature comparison. BMC Nephrol.

[7] Liu Q, Celis-Morales C, Lees JS (2025). Discordance between cystatin C-based and creatinine-based estimated glomerular filtration rate and mortality in the general population. Clin Chem.

[8] Gosmanova EO, Baumgarten DA, O'Neill WC (2009). Acute kidney injury in a patient with unilateral ureteral obstruction. Am J Kidney Dis.

[9] Jiang R, Huang Y, Zeng M, Xing C, Mao H, Wu B (2023). A marked elevation in serum creatinine/cystatin C ratio may indicate pseudo-acute kidney injury due to urinary ascites: a case report and literature review. BMC Nephrol.

[10] Zaid M, Al Mohdi OA, Alhayek R, Alsadi AM, Yusuf FA, Saeedi Y (2026). Acute obstructive uropathy complicated by spontaneous fornix rupture and hemorrhagic urinoma. Cureus.

[11] Larsson A, Malm J, Grubb A, Hansson LO (2004). Calculation of glomerular filtration rate expressed in mL/min from plasma cystatin C values in mg/L. Scand J Clin Lab Invest.

[12] Levey AS, Stevens LA, Schmid CH (2009). A new equation to estimate glomerular filtration rate. Ann Intern Med.

[13] Shekar A, Dumra A, Reddy D, Patel H (2022). An interesting presentation of invasive bladder carcinoma as pseudo renal failure. J Bras Nefrol.

[14] Inker LA, Schmid CH, Tighiouart H (2012). Estimating glomerular filtration rate from serum creatinine and cystatin C. N Engl J Med.

[15] Inker LA, Eneanya ND, Coresh J (2021). New creatinine- and cystatin C-based equations to estimate GFR without race. N Engl J Med.

[16] Pottel H, Delanaye P, Schaeffner E (2017). Estimating glomerular filtration rate for the full age spectrum from serum creatinine and cystatin C. Nephrol Dial Transplant.

